# *Origanum heracleoticum* Essential Oils: Chemical Composition, Phytotoxic and Alpha-Amylase Inhibitory Activities

**DOI:** 10.3390/plants12040866

**Published:** 2023-02-14

**Authors:** Giuseppe Amato, Lucia Caputo, Rosaria Francolino, Mara Martino, Vincenzo De Feo, Laura De Martino

**Affiliations:** Department of Pharmacy, University of Salerno, Via San Giovanni Paolo II, 132, 84084 Fisciano, Italy

**Keywords:** *Origanum heracleoticum* L., essential oil, phytotoxic activity, amylase

## Abstract

Many studies have demonstrated the herbicidal effects of several essential oils and their possible use as substitutes for chemical herbicides. Several enzymes play a very significant role in seed germination: among these, α-amylase could be involved in essential oil phytotoxic processes. The aims of this study were to compare the chemical composition of the essential oils of two ecotypes of *O. heracleoticum* growing in Cilento (Southern Italy) and to study their possible use as natural herbicide using *Raphanus sativus*, *Sinapis arvensis* and *Lolium multiflorum* seeds. Moreover, a possible inhibitory activity on the α-amylase enzyme extracted from germinating seeds was evaluated as a possible mechanism of action. Both oils, characterized by GC-MS, belonged to a carvacrol chemotype. The alpha-amylase activity was determined using DNSA (dinitrosalicylic acid) assay quantifying the reducing sugar produced. Furthermore, the essential oils demonstrated phytotoxicity at the highest dose tested, and an inhibitory effect on α-amylase, probably correlated with the phytotoxic effects, was registered. The oils showed interesting phytotoxic and alpha-amylase inhibitory activities, which deserve to be further investigated.

## 1. Introduction

Crop yield is reduced by nearly 34% due to weeds alone [1]. The total value of the world’s agrochemical market is between USD 31 and 35 billion, and herbicides account for 48% of the total, followed by insecticides, accounting for 25%, and fungicides, at 22% [2].

Moreover, the extensive use of synthetic herbicides causes human health and environmental problems. Moreover, some weeds are developing resistance to classical herbicides. Essential oils possess various biological properties and could represent a potential candidates for weed management [3,4]. Even if EOs present advantages, they also present some limitations [5]. For example, beyond being easily extractable and generally eco-friendly, they are also biodegradable and easily catabolized in the environment [6]; moreover, they do not persist for long in soil or water [7]. On the other hand, EOs have an excessively short environmental half-life, and patent protection may be limited [5]. Plants in Lamiaceae family possess EOs with potential herbicidal activity [8]. The genus *Origanum*, widely distributed in the Mediterranean region, belongs to Lamiaceae family, and comprises 42 species with flowers and leaves with a characteristic odor [9,10]. Generally, the genus is constituted by perennial herbs, with creeping roots, erect and woody stems, with sub-sessile to petiolate and hairy leaves [11]. Among traditional medical uses of the *Origanum* species, the treatment of abdominal pain, toothache, headache, diabetes, infections of the urinary tract, gastrointestinal and pulmonary diseases was reported [12,13,14]. Previous studies have shown antibacterial, antifungal, anticancer, antidiabetic, antinociceptive, insecticidal and phytotoxic activities for some *Origanum* species [12,13,14,15,16,17,18,19,20,21]. Interestingly, their essential oils (EOs) also showed ferric reducing and antioxidant properties [22]. The bioactivity of the EOs from *Origanum* species has been primarily attributed to the presence of carvacrol and/or thymol, their main and the most active components [17,18,23], although a recent study carried out on *Origanum vulgare* subsp. *vulgare* EO from different parts of the cultivated and wild plant showed that caryophyllene oxide was the main compound in all the essential oil samples. Moreover, EOs from wildflowers showed the highest antioxidant activity, with an EC_50_ value of 4.78 mg/mL [24]. *O. heracleoticum* L., also known as *Origanum vulgare* subsp. *viridulum* (Martrin-Donos) Nyman [25], and with the name of ‘Greek oregano’, has been used in traditional herbal medicine in the treatment of cough and toothache [26].

Few reports on chemical composition and biological activities of the EO of *O. heracleoticum* are available in the literature [17,27,28,29,30]. Napoli and coworkers reported data on variation in the aromatic profile of essential oils from a 3-year cultivation trial of *Origanum vulgare* subsp. *viridulum* grown in Sicily. In these EOs, carvacrol was never greater than 1% of the total composition [27]. A study on *Origanum vulgare* subsp. *hirtum* from Turkey rich in carvacrol and γ-terpinene showed that this EO could be used to combat increasing resistance to antibiotics and can be an alternative in the food preservation [28].

Recent studies have reported its antimicrobial activity against the growth of *Escherichia coli* and *Pseudomonas aeruginosa* clinical strains and against postharvest phytopathogens [17,29,30].

Moreover, the EO was active against the germination and initial radicle growth of *Raphanus sativus* L. (radish), *Lactuca sativa* L. (lettuce), *Lepidium sativum* L. (garden cress) and *Solanum lycopersicum* L. (tomato) [17].

Although many studies have focused on assessing the herbicidal potential of essential oils and their main constituents [15,31,32,33], few studies have reported a possible correlation between some essentials oil phytotoxic activity and α-amylase activity [34,35,36]. In plant seeds, the amount of readily utilizable sugar is very limited, and starch is the main reserve carbohydrate [37]. Alpha-amylase is considered to be the enzyme that plays the most important role in degradation of reserve carbohydrate to soluble sugars during germination [38].

Therefore, a possible reduction in α-amylase exercised by essential oils could inhibit respiratory metabolism, allowing the germination of plant seeds [36].

The aims of this study were: (i) to determine the phytochemical profile of the EOs from two ecotypes of *O. heracleoticum* from Cilento, Southern Italy; (ii) to evaluate their potential phytotoxic effects against radicle elongation and germination of *Raphanus sativus*, *Sinapis arvensis* L. and *Lolium multiflorum* Lam. seeds; and (iii) to find a possible correlation between phytotoxic activity and α-amylase activity evaluating as a possible mechanism of action an inhibition of the α-amylase extracted from germinating seeds.

## 2. Results and Discussion

### 2.1. Chemical Composition

The hydrodistillation of the aerial parts of *O. heracleoticum* provided pale yellow EOs with a weight of 6.54 g for OHW, with a yield of 1.1%, and 5.07 g for OHR, with a yield of 0.6%. Table 1 reports the compounds of two essential oils in order of their elution on a HP-5MS column.

**Figure 1:**
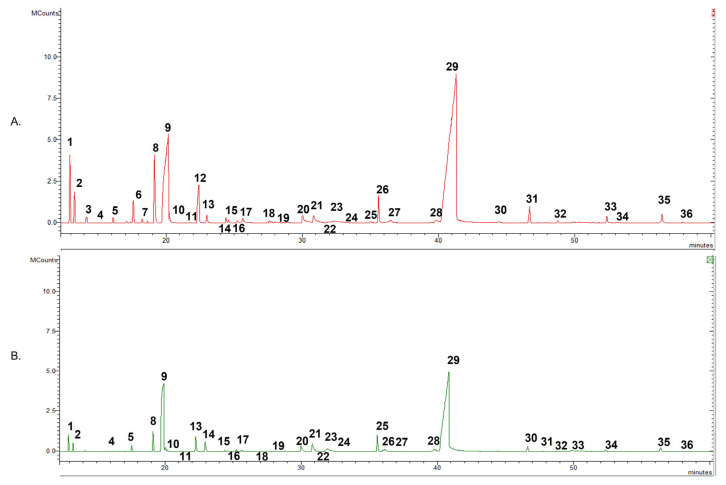
The gas chromatography (GC) chromatograms of *O. heracleoticum* essential oils: (**A**) OHR; (**B**) OHW. The numbers in the chromatograms indicate the compounds reported in Table 1.

In total, 38 compounds were annotated: 35 for OHW and 38 for OHR, accounting respectively for 98.3%and 97.5% of the total EOs. Carvacrol was the main component for both EOs. Figure 1 presents the GC profile of two essential oils.

Oxygenated monoterpenes were the main constituents of the EOs, comprising 62.6% of OHW and 61.3% of OHR. Monoterpene hydrocarbons represented 32.6% of OHW and 32.3% of OHR. Although the percentages of monoterpene hydrocarbons were similar, several differences in both the amount and the number of components were registered. In fact, in OHW EO, a higher amount of *p*-cymene than in OHR EO was registered (22.0 vs. 7.0%). On the other hand, the OHR EO presented greater amounts of α-thujene (3.1 vs. 1.4%), α-pinene (1.3 vs. 0.7%) and β-pinene (1.4 vs. 0.7%). δ-3-carene, α-Phellandrene, and β-ocimene were present only in the EO of OHR, with percentages of 0.1, 0.2 and 7.7%, respectively.

Sesquiterpenes also presented slight differences between the two EOs. In fact, sesquiterpenes hydrocarbons were more abundant in OHR (2.2%) than in OHW (1.5%), while the opposite was the case for oxygenated sesquiterpenes. Even for sesquiterpenes, some quantitative differences were registered: aceto-vanillone was present in greater amounts in OHW (1.5 vs. 0.2%), while caryophyllene oxide was present in greater amounts in OHR (0.9 vs. 0.1%).

According to Russo and coworkers [41], four chemotypes of oregano (thymol, carvacrol thymol/carvacrol and carvacrol/thymol) were found. Vokou and coworkers [42] studied the geographic variation of greek oregano and they found that some EOs could be considered constituted of only carvacrol (up to 90%) or thymol (up to 90%). In some other cases, carvacrol was the main component, and thymol was not present in detectable amounts, as in the samples studied here.

Kokkini and coworkers [43] reported the chemical compositions of some Greek oregano EOs from three distinct geographic areas of Greece: the EOs presented as major components either *p*-cymene (ranging from 17.3 to 26.9%) or carvacrol (57.4–69.6%). Additionally, OHW and OHR presented carvacrol and *p*-cymene as major components. Instead, the essential oil of *O. heracleoticum* L. analyzed by Dzamic and collaborators [44], presented carvacrol (65.12%) and thymol (14.84%) as major constituents. Baycheva and Dobreva [45], in agreement with the results reported here, found, in a Bulgarian *O. heracleoticum* L. EO, carvacrol and *p*-cymene as the main constituents. *p*-Cymene was reported as a precursor of thymol: by aromatization of *γ-*terpinene, *p*-cymene was produced; subsequentl, *p*-cymene was hydroxylated to thymol [46]. Additionally, the biosynthetic pathway of carvacrol could be related to *p*-cymene: the desaturation of *γ*-terpinene to *p*-cymene followed by hydroxylation at the C-2 of the ring resulted in carvacrol [46]. The chemical analysis of our EOs confirmed the presence of compounds involved in the same biosynthetic pathway [46,47].

Several factors, such as plant metabolism, climatic factors, and the secretory activity of glandular hairs, could affect secretion of essential oils, and thus their chemical composition, modifying the amounts of the main components linked each other by the same biosynthetic pathway [47].

### 2.2. Phytotoxic Activity and α-Amylase Activity of Germinating Seeds

The EOs were studied for their activity against germination and radicle elongation of *R. sativus*, *S. arvensis* and *L. multiflorum* (Table 2 and Table 3), showing a similar phytotoxic activity.

In fact, they inhibited the germination of *S. arvensis* seeds at 1000 and 500 µg/mL, while this inhibitory activity was also exhibited by OHR at 250 µg/mL. Moreover, both EOs were able to inhibit radicle elongation of *S. arvensis* at the highest dose used (1000 µg/mL).

The EOs inhibited the radical growth of *R. sativus* at 1000 and 500 µg/mL. OHW EO inhibited *R. sativus* germination at all doses tested, whereas OHR EO was active only at 500 and 250 µg/mL.

*L. multiflorum* seed was the most sensitive; in fact, germination and radical growth were completely inhibited at 1000, 500 and 250 µg/mL.

Finally, with respect to the number of germinated seeds, the EOs seemed not to be selective against the weed *L. multiflorum*; rather, upon radicle elongation, they were selective at a dose of 250 µg/mL for the tested weed.

Even if *O. heracleoticum* is one of the less-studied species, these results corroborated those reported in several studies in which a great phytotoxic activity of different essential oils from *Origanum* genus was reported [9,15,17,18,48,49].

Mancini and coworkers [49] evaluated the phytotoxic effects of three EOs of *O. vulgare* L. ssp *hirtum* (Link) Ietsw. from plants of three different localities in southern Italy against growth of *Raphanus sativus*, *Lepidium sativum* L., *S. arvensis* and *Phalaris canariensis* L. (canary cress): the EOs mainly inhibited the germination and the radicle elongation of *P. canariensis*.

Subsequently, Elshafie et al. [50] and Della Pepa et al. [17] studied the phytotoxicity of EOs of *O. vulgare* ssp. *hirtum* and *O. heracleoticum* L., and of carvacrol, their main component: the EOs, and mainly carvacrol, showed a remarkable phytotoxic activity against *Lactuca sativa* L. (lettuce), *L. sativum* and *Solanum lycopersicum* L. (tomato) seeds.

Previously, our research group also studied the phytotoxic effects the EO of a carvacrol chemotype *Origanum vulgare* L. [15,18]: the EO showed significant inhibitory effects on the seeds of *R. sativus*, *L. sativum* and *L sativa* [15] and on the seeds of invasive *Solidago canadensis* L. (canadian goldenrod) [18].

Although these studies carried out on different species of *Origanum* genus reported a similar phytotoxic activity to the studied samples, it is important to highlight that the chemical composition is different with respect to compounds present in lower concentrations than carvacrol. *p*-Cymene, *α-*terpinene and *γ-*terpinene were present in the studied samples in percentages between 7.0 and 22.0% for *p*-Cymene, 3.4 and 3.5% for α-terpinene, and 2.0 and 3.8% for *γ*-terpinene. In contrast, in other research [15,17,18,49,50], these compounds were present in trace amounts or with lower concentrations than in the studied EOs.

The phytotoxic activity of our EOs could be related to the presence of a substantial amount of oxygenated compounds, in particular of carvacrol, also reported in literature for its phytotoxic effect [49,50,51]. Monoterpene hydrocarbons, such as *p*-cymene and *γ*-terpinene, and oxygenated monoterpenes, such as carvacrol, had phytotoxic effects that may cause anatomical and physiological alterations in plant seedlings leading to accumulation of lipid globules in the cytoplasm, reduction in some organelles such as mitochondria, probably linked to block of DNA synthesis or breaking of membranes [15,17,21].

In this paper, two crops and one weed species were tested: weeds represent one of the most severe problems in agricultural practices nowadays together with the environmental problems caused by synthetic herbicides. Damage due to invasive plants is one of the principal restricting factors in basic crops worldwide [52]; developing a selective natural herbicide that does not cause chemical pollution, thus protecting the environment and sustaining biodiversity, is actually difficult, even if some data are promising.

The major source of energy for seed germination was the degradation of endosperm starch. Different enzymes played an important role in the starch hydrolysis, including α and β-amylases, debranching enzyme, and α-glucosidase [53].

In this study, the maltose produced was evaluated after reaction with α-amylase present in extracts of 5-day-old seedlings of *S. arvensis*, *R. sativus* and *L. multiflorum*, after the treatment with the two EOs. The results were reported in Table 4.

The data showed that the OHW EO significantly reduced the quantity of maltose produced of *S. arvensis* and *L. multiflorum* seedling extracts at 1000, 500 and 250 µg/mL, and at 1000 and 500 µg/mL in the case of *L. multiflorum* seeds.

The OHR reduced the amount of maltose produced in all three seeds: in *S. arvensis* and *R. sativus* seedlings, at doses of 1000, 500 and 250 µg/mL; in *L. multiflorum* seeds at the highest dose tested (1000 µg/mL).

The enzymatic activity in the extracts of the seedlings was calculated to highlight its possible involvement in the observed phytotoxic activity (Table 2 and Table 3). The results (Table 5) showed that essential oils significantly inhibited the α-amylase present in all seedlings and at all tested concentrations. The results of the enzymatic activity confirm the data obtained on the number of germinated seeds (Table 3).

The amylase inhibition could be one of the molecular processes involved in the phytotoxicity of the two EOs. No previous studies on *Origanum* species reported a possible correlation between phytotoxicity and α-amylase inhibition, even if few previous studies reported a good α-amylase inhibitory activity of EOs from *O. vulgare* subsp. *vulgare*, *O. vulgare* subsp. *hirtum* or *O. vulgare* subsp *glandulosum* Desf. [54,55].

## 3. Materials and Methods

### 3.1. Chemicals

Aromadendrene, Borneol, *α*-Bisabolol, Camphene, Carvacrol, Caryophyllene oxide, 1,8-Cineole, *α-*Humulene, Limonene, Linalool, *(E)-β*-Ocimene, *α-*Phellandrene, *α-*Pinene, *β-*Pinene, Sabinene, *α-*Terpinene, *γ-* Terpinene, Terpinen-4-ol, *α-*Terpineol, *α-*Thujene, Thymol, soluble starch, DNSA (dinitrosalicylic acid), were purchased from Sigma Aldrich (Milan, Italy). Sodium tartrate, sodium potassium tartrate, sodium acetate were bought from Merck (Darmstadt, Germany).

### 3.2. Plant Material

Representative homogeneous samples of two different ecotypes of *O. heracleoticum* were collected in September 2021, in Atena Lucana, Salerno province, Southern Italy. In particular, one *O. heracleoticum* ecotype was with white flowers (OHW) and the other *O. heracleoticum* ecotype was with red flowers (OHR). The ecotypes were identified by Prof. V. De Feo, based on *Flora d’Italia* [56], and voucher specimens have been deposited in the herbarium of the Medical Botany Chair of University of Salerno.

### 3.3. Isolation of the Volatile Oil

Six hundred grams of dried aerial parts of *O. heracleoticum* ecotypes were ground in a Waring blender and then subjected to hydrodistillation for 3 h, according to the standard procedure described in the *European Pharmacopoeia* [57]. The oils were solubilized in *n*-hexane, filtered over anhydrous sodium sulphate, and stored under N_2_ at +4 °C in the dark until tested and analyzed.

### 3.4. Analysis of the Essential Oils

Gas chromatography was conducted on a Perkin-Elmer Sigma-115 gas chromatograph (Perkin Elmer, Waltham, MA, USA) endowed with a flame ionization detector (FID) and a data handling processor. The separation was achieved with a HP-5 MS fused-silica capillary column (30 m × 0.25 mm i.d., 0.25 µm film thickness, Agilent, Agilent, Santa Clara, CA, USA). Column temperature: 40 °C, with 5 min initial hold, and then to 270 °C at 2 °C/min, 270 °C (20 min); injection means, without split (1 L of a 1:1000 n-hexane solution); injector and detector temperatures were fixed to 250 °C and 290 °C, respectively. The analysis was also conducted with a fused silica HP Innowax polyethylene glycol capillary column (50 m × 0.20 mm i.d., 0.25 µm film thickness, Agilent, Santa Clara, CA, USA). Column temperature: 60 °C (5 min) and then 10 °C/min, 220 °C (10 min). In both conditions, helium was employed as the carrier gas (1.0 mL/min).

GC/MS analyses were conducted on an Agilent 6850 Ser. II apparatus (Agilent, Santa Clara, CA, USA), equipped with a DB 5 fused silica capillary column (30 m × 0.25 mm i.d., 0.33 µm film thickness, connected to an Agilent Mass Selective Detector MSD 5973; ionization energy voltage 70 eV; electron multiplier voltage energy 2000 V. Mass spectra (MS) were acquired between 40 and 500 amu; scan time, 5 scans/s.

The gas chromatographic features were as indicated in the preceding paragraph; transferline temperature, 295 °C.

**Figure 2:**
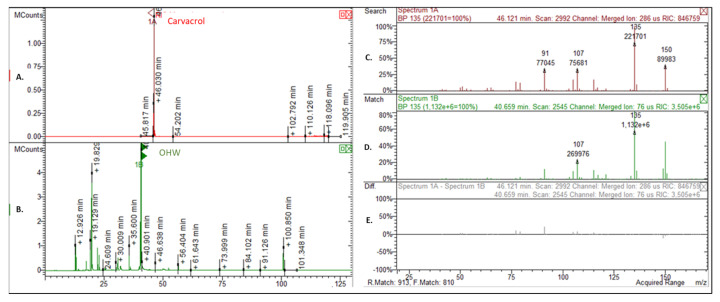
Example of method used for essential oils compound annotation: (**A**) standard GC chromatogram; (**B**) essential oil GC chromatogram; (**C**) standard mass spectrum; (**D**) corresponding mass spectrum obtained in the essential oil GC chromatogram; (**E**) differences between two mass spectrum.

Annotation of essential oil components was performed by comparing their mass spectra with those reported in the literature [39,40,58,59,60] and confirmed by comparing their experimentally determined Kovats retention indices (KI). In order to calculate the relative retention indices (KI), a homologous series of n-alkanes (C_10_–C_35_) were used as a reference. Further annotation was performed by comparing their mass spectra with those of authentic compounds available in our laboratory and injected under the same conditions (Figure 2). The components relative concentrations were obtained by peak area normalization. Response factors were not considered.

### 3.5. Phytotoxic Activity and Determination of α-Amylase Activity of Germinating Seeds

#### 3.5.1. Phytotoxic Activity

The phytotoxic activity was studied on radicle elongation and germination of the seeds of two crops *Raphanus sativus* L. (radish), *Sinapis arvensis* L. (wild mustard), and one weed *Lolium multiflorum* Lam. (Italian ryegrass). *R. sativus* seeds were purchased from Blumen group s.r.l. (Bologna, Italy); *L. multiflorum* seeds were bought from Fratelli Ingegnoli s.p.a. (Milano, Italy); *S. arvensis* seeds were picked from wild populations in Sidi Ismail, Beja, Tunisia on June 2021. These seeds, usually employed in phytotoxic tests, are easily germinable and are well known from a histological point of view. The seeds were sterilized in 95% ethanol for 15 s and sown in Petri dishes (Ø = 90 mm), on three layers of Whatman filter paper, impregnated with distilled water (7 mL, control) or the essential oil solution (7 mL), at different doses. The germination conditions were 20 ± 1 °C, with natural photoperiod. The EOs, solubilized in water–acetone mixture (99.5:0.5 *v*/*v*) to increase their solubility, were tested at the doses of 1000, 500, 250, 100 μg/mL. No differences in activity were registered between controls performed with water–acetone mixture and controls with water alone. Seed germination was observed in Petri dishes every 24 h. A seed was considered germinated when the protrusion of the root became evident [61]. After 120 h for *R. sativus* and *S. arvensis* seeds and 168 h for *L. multiflorum* seeds, the radicle lengths were determined and are expressed in cm. Each determination was conducted three times, using Petri dishes with 10 seeds inside.

#### 3.5.2. Determination of α-Amylase Activity of Germinating Seeds

The determination of α-amylase activity was carried out by quantifying the reducing sugar (maltose equivalent) liberated by the treated seedlings under the assay conditions described in the previous paragraph. Aqueous extracts of seedlings were prepared using the protocol of Elkhalifa and Bernhardt [62] with slight modification. Briefly, 2 g of the germinated seeds were ground using mortar and pestle with 10 mL of distilled water and macerated for 1 h at 25 °C with occasional shaking and then centrifuged at 6000 g for 10 min. The supernatant taken for the determination of α-amylase activity. All the preparations were carried out at 4 °C.

α-Amylase activity was determined using the method of Bernfeld [63] with slight modification. One hundred microliters of supernatant was added to 300 µL of 20 mM sodium phosphate buffer (pH = 6.9) and 180 µL of 1% soluble starch solution. The mixture was incubated at 37 °C for 10 min. One hundred eighty microliters of DNSA solution was added to the mixture and boiled in a water bath at 100 °C for 10 min. Then, the solution was cooled by adding 600 µL of distilled water. Absorbance of the solution was read at 540 nm in a UV Spectrophotometer (Thermo Fischer Scientific, Vantaa, Finland). The maltose produced by α-amylase seedling extract was estimated from a standard curve plotted using the maltose linear regression equation Y = 1.4929x + 0.0037; R^2^ = 0.9997, and the results are expressed as equivalent maltose per gram of essential oil.

The enzyme activity was estimated using the following formula, and the results are expressed as µmol min^−1^ L^−1^:

$$\;\mathrm{Enzyme}\;\mathrm{activity}=\frac{g\;\mathrm{maltose}\;\mathrm{produced}}{\mathrm{maltose}\;\mathrm{MW}\;L\;\min}$$

where L indicates the volume in liters of α-amylase and min was the time of the reaction.

### 3.6. Statistical Analysis

Each experiment was carried out in triplicate. Statistical analysis was conducted using the GraphPad Prism 6.0 software (GraphPad Software Inc., San Diego, CA, USA) with two-way ANOVA analysis followed by the Dunnett’s multiple comparisons test. Data were considered statistically significant at the level of *p* < 0.05.

## 4. Conclusions

This study analyzed the chemical profile and phytotoxic activity of the EOs of two ecotypes of *O. heracleoticum* from Cilento, which have never been analyzed before. Moreover, the possible involvement of α-amylase inhibition as a cause of essential oil phytotoxic activity was evaluated. The results contribute to the elucidation of the importance of essential oils in agricultural practices: the phytotoxic effects of these two EOs, correlated with their ability to inhibit the α-amylase, suggests a basis for future research aiming to evaluate their possible use as a potential natural herbicide. Plants could be an important resource for developing new herbicides: a high eco-compatibility and the possibility of avoiding the indiscriminate use of chemical herbicides, unsafe for both the environment and human health, as largely demonstrated in the literature, are among the advantages of the use of EOs in agriculture. Moreover, the correlation of phytotoxic activity with α-amylase inhibitory activity could ease the identification of possible natural herbicide.

## Figures and Tables

**Table 1:** Chemical composition of the EOs of OHW and OHR.

A. HP-5MS Column						
		OHW	OHR	KI ^a^	KI ^b^	Identification ^e^
1	α-Thujene	1.4	3.1	924	930	1, 2, 3
2	α-Pinene	0.7	1.3	932	939	1, 2, 3
3	Camphene	0.2	0.4	949	954	1, 2, 3
4	Sabinene	0.1	0.2	971	975	1, 2, 3
5	β-Pinene	0.7	1.4	976	979	1, 2, 3
6	α-Phellandrene	-	0.2	1007	1002	1, 2, 3
7	δ-3-Carene	-	0.1	1009	1011	1, 2, 3
8	α-Terpinene	2.0	3.8	1014	1017	1, 2, 3
9	***p*** **-Cymene**	**22.0**	**7.0**	1023	1024	1, 2, 3
10	Limonene	1.0	3.4	1028	1029	1, 2, 3
11	1,8-Cineole	t	t	1031	1031	1, 2, 3
12	*(E)-*β-Ocimene	-	7.7	1043	1050	1, 2, 3
13	γ- Terpinene	3.5	3.4	1056	1059	1, 2, 3
14	*cis*- Sabinene hydrate	0.5	0.5	1065	1070	1, 2, 3
15	Terpinolene	0.2	0.2	1084	1088	1, 2
16	*trans*-Sabinene hydrate	0.2	0.2	1079	1098	1, 2, 3
17	Linalool	0.7	0.7	1099	1096	1, 2, 3
18	*allo*-Ocimene	0.1	0.1	1140	1132	1, 2, 3
19	*dehydro*-Linalool	0.5	0.5	1150	1135	1, 2
20	Borneol	1.1	1.1	1071	1169	1, 2, 3
21	Terpinen-4-ol	1.0	1.0	1180	1177	1, 2, 3
22	*meta*-Cymen-8-ol	0.3	0.3	1198	1179	1, 2, 3
23	α-Terpineol	0.5	0.5	1180	1188	1, 2, 3
24	*cis*-Dihydrocarvone	0.2	0.2	1190	1192	1, 2, 3
25	Thymol methyl ether	1.6	1.6	1240	1235	1, 2
26	*p*- Cymenene	0.2	0.2	1211	1250	1, 2, 3
27	Thymoquinone	0.5	0.5	1251	1252	1, 2
28	Thymol	0.5	0.5	1292	1290	1, 2, 3
29	**Carvacrol**	**54.9**	**54.1**	1301	1299	1, 2, 3
30	*(E)-*Caryophyllene	1.0	1.1	1415	1419	1, 2, 3
31	Aromadendrene	0.1	t	1420	1441	1, 2, 3
32	α-Humulene	0.1	0.2	1435	1454	1, 2, 3
33	Acetovanillone	1.5	0.2	1446	1482	1, 2
34	Germacrene A	0.4	0.9	1489	1509	1, 2
35	Caryophyllene oxide	0.1	0.9	1552	1583	1, 2, 3
36	*trans*-Dihydrocarvone	0.3	0.3	1606	1627	1, 2, 3
37	α-Bisabolol	0.3	0.3	1670	1685	1, 2, 3
	**Total**	**98.3**	**97.5**			
	Monoterpene hydrocarbons	32.3	32.6			
	Oxygenated monoterpenes	62.6	61.3			
	Sesquiterpene hydrocarbons	1.5	2.2			
	Oxygenated sesquiterpenes	1.9	1.4			
B. HP Innowax column						
		KI ^c^		KI ^d^		**Identification ^e^**
1	α-Pinene	1015		1015		1, 2, 3
2	α-Thujene	1021		1221		1, 2, 3
3	Camphene	1052		1075		1, 2, 3
4	β-Pinene	1087		1087		1, 2, 3
5	Sabinene	1106		1112		1, 2, 3
6	δ-3-Carene	1132		1153		1, 2, 3
7	α-Phellandrene	1151		1160		1, 2, 3
8	α-Terpinene	1168		1166		1, 2, 3
9	1,8-Cineole	1211		1210		1, 2, 3
10	Limonene	1212		1217		1, 2, 3
11	*(E)-*β-Ocimene	1235		1248		1, 2, 3
12	γ- Terpinene	1239		1253		1, 2, 3
13	***p*** **-Cymene**	1263		1274		1, 2, 3
14	Terpinolene	1269		1278		1, 2
15	*allo*-Ocimene	1390		1382		1, 2, 3
16	*p*- Cymenene	1417		1414		1, 2, 3
17	*cis*- Sabinene hydrate	1500		-		1, 2, 3
18	*trans*-Sabinene hydrate	1547		1546		1, 2, 3
19	Linalool	1557		1551		1, 2, 3
20	Terpinen-4-ol	1595		1595		1, 2, 3
21	Thymol methyl ether	1610		1604		1, 2
22	*(E)-*Caryophyllene	1616		1612		1, 2, 3
23	*dehydro*-Linalool	1617		1617		1, 2
24	*cis*-Dihydrocarvone	1620		-		1, 2, 3
25	*trans*-Dihydrocarvone	1624		1627		1, 2, 3
26	Aromadendrene	1635		1637		1, 2, 3
27	α-Terpineol	1642		1662		1, 2, 3
28	α-Humulene	1679		1671		1, 2, 3
29	Borneol	1698		1715		1, 2, 3
30	Germacrene A	1712		1747		1, 2
31	*meta*-Cymen-8-ol	1845		1849		1, 2, 3
32	α-Bisabolol	2021		2232		1, 2, 3
33	Thymoquinone	2180		-		1, 2
34	Thymol	2200		2172		1, 2, 3
35	**Carvacrol**	2242		2225		1, 2, 3
36	Caryophyllene oxide	2500		1989		1, 2, 3
37	Acetovanillone	2667		2676		1, 2

^a^ Kovats index on a HP-5MS column; ^b,d^ Kovats index from [39,40]. ^c^ Kovats index on a HP Innowax column; ^e^ identification method: 1 = Kovats index; 2 = comparison of mass spectra; 3 = Co-injection with standard compounds. *t* = traces < 0.05%; - = absent.

**Table 2:** Germination of *R. sativus*, *S. arvensis* and *L. multiflorum* seeds treated with different doses of EOs. The measurement was carried out 120 h after sowing. Results are expressed as the mean of three experiments ± SD.

	Number of Germinated Seeds		
	*S. arvensis*	*R. sativus*	*L. multiflorum*
H_2_O	9.3 ± 1.1	6.0 ± 1.7	7.3 ± 0.6
OHW			
1000 µg/mL	0.0 ± 0.0 ****	0.0 ± 0.0 ***	0.0 ± 0.0 ****
500 µg/mL	3.0 ± 1.4 ****	2.0 ± 0.0 **	0.0 ± 0.0 ****
250 µg/mL	6.5 ± 0.7 *	1.0 ± 0.0 ***	0.0 ± 0.0 ****
100 µg/mL	10.0 ± 0.0	3.0 ± 1.0 *	8.0 ± 1.7
OHR			
1000 µg/mL	0.0 ± 0.0 ****	0.0 ± 0.0 **	0.0 ± 0.0 ****
500 µg/mL	0.5 ± 0.7 ****	0.0 ± 0.0 **	0.0 ± 0.0 ****
250 µg/mL	1.0 ± 0.0 ****	2.0 ± 2.8	0.0 ± 0.0 ****
100 µg/mL	8.7 ± 1.2	5.3 ± 1.5	8.0 ± 1.0

* *p* < 0.05; ** *p* < 0.01; *** *p* < 0.001; **** *p* < 0.0001 compared with H_2_O.

**Table 3:** Phytotoxic activity of the essential oils of *O. heracleoticum* against radicle elongation of *R. sativus*, *S. arvensis* and *L. multiflorum* 120 h after sowing. Results are expressed as the mean of three experiments ± SD.

	Radicle Elongation (cm)		
	*S. arvensis*	*R. sativus*	*L. multiflorum*
H_2_O	1.9 ± 1.0	2.4 ± 1.0	4.9 ± 1.7
OHW			
1000 µg/mL	0.0 ± 0.0 *	0.0 ± 0.0 ***	0.0 ± 0.0 ***
500 µg/mL	0.3 ± 0.2	1.2 ± 0.2 *	0.0 ± 0.0 ***
250 µg/mL	1.4 ± 0.6	1.5 ± 0.1	0.0 ± 0.0 ***
100 µg/mL	2.4 ± 1.2	1.8 ± 0.4	1.8 ± 0.7
OHR			
1000 µg/mL	0.0 ± 0.0 *	0.0 ± 0.0 ***	0.0 ± 0.0 ***
500 µg/mL	0.5 ± 0.1	0.0 ± 0.0 ***	0.0 ± 0.0 ***
250 µg/mL	1.7 ± 1.4	1.4 ± 0.3	0.0 ± 0.0 ***
100 µg/mL	2.7 ± 1.5	2.2 ± 0.8	2.0 ± 0.7

* *p* < 0.05; *** *p* < 0.001 compared with H_2_O.

**Table 4:** Maltose equivalent content of 5 days old seedlings of *S. arvensis*, *R. sativus* and *L. multiflorum* treated with different concentrations of the EOs. Data are expressed as the average of three experiments ± SD.

	Maltose Equivalent (mg/g)		
	*S. arvensis*	*R. sativus*	*L. multiflorum*
H_2_O	1.44 ± 0.44	2.08 ± 0.78	10.75 ± 1.96
OHW			
1000 µg/mL	0.42 ± 0.06 **	1.22 ± 0.38	7.28 ± 0.02 **
500 µg/mL	0.69 ± 0.10 *	0.75 ± 0.50	8.05 ± 0.39 *
250 µg/mL	0.67 ± 0.20 *	0.82 ± 0.54	9.64 ± 0.47
100 µg/mL	0.87 ± 0.37	1.82 ± 0.89	9.14 ± 0.51
OHR			
1000 µg/mL	0.47 ± 0.02 *	0.69± 0.17 **	7.68 ± 0.64 *
500 µg/mL	0.34 ± 0.20 *	0.87 ± 0.38 *	8.12 ± 0.21
250 µg/mL	0.45 ± 0.40 *	0.71 ± 0.49 **	8.08 ± 0.35
100 µg/mL	2.28 ± 0.58	1.56 ± 0.07	8.69 ± 1.73

* *p* < 0.05; ** *p* < 0.01 compared with H_2_O.

**Table 5:** α-amylase activity of *S. arvensis, R. sativus,* and *L. multiflorum* seedlings at 5 days old.

	α-Amylase Activity (µmol min^−1^ L^−1^)		
	*S. arvensis*	*R. sativus*	*L. multiflorum*
H_2_O	421.0 ± 7.8	608.1 ± 5.9	3143.2 ±59.7
OHW			
1000 µg/mL	122.8 ± 5.6 ****	356.7 ± 5.2 ****	212.8 ± 9.7 ****
500 µg/mL	201.7 ± 3.4 ****	219.2 ± 6.7 ****	235.4 ± 8.6 ****
250 µg/mL	195.9 ± 5.2 ****	239.7 ± 7.9 ****	281.8 ± 6.9 ****
100 µg/mL	254.3 ± 6.7 ****	532.1 ± 5.1 ****	267.2 ± 7.8 ****
OHR			
1000 µg/mL	137.4 ± 3.2 ****	201.7 ± 4.5 ****	224.5 ± 9.7 ****
500 µg/mL	99.0 ± 8.9 ****	254.3 ± 5.2 ****	237.4 ± 8.4 ****
250 µg/mL	131.5 ± 3.2 ****	207.6 ± 6.4 ****	236.2 ± 7.3 ****
100 µg/mL	666.6 ±7.5 ****	456.1 ± 7.3 ****	254.1 ± 7.1 ****

**** *p* < 0.0001 compared with H_2_O.

## Data Availability

The EOs studied are available on request from the corresponding author.
